# How primary care physicians view kidney supportive care

**DOI:** 10.1093/ckj/sfad305

**Published:** 2023-12-27

**Authors:** Cynthia C Lim, Zhong Hong Liew, Li Choo Ng, Jason Choo, Jia Liang Kwek

**Affiliations:** Department of Renal Medicine, Singapore General Hospital, Singapore; Department of Renal Medicine, Singapore General Hospital, Singapore; Department of Renal Medicine, Singapore General Hospital, Singapore; Specialty Nursing, Singapore General Hospital, Singapore; Department of Renal Medicine, Singapore General Hospital, Singapore; Department of Renal Medicine, Singapore General Hospital, Singapore

To the Editor,

The prevalence of chronic kidney disease (CKD) has increased exponentially in Asian populations with a high burden of metabolic risk factors such as diabetes and hypertension [1]. Accordingly, the number of individuals with advanced CKD who are not on dialysis (CKD5ND) due to patient preference or barriers to dialysis is also expected to rise. Thus, primary care providers (PCPs) may also encounter these individuals more frequently in their practice. It is timely that the article ‘Factors that influence the selection of conservative management for end-stage renal disease—a systematic review’ highlighted that primary care providers (PCPs) can play an important role as patient advocates in shared decision-making and continuing care for these individuals [2].

To assess the comfort of our PCPs in managing individuals with CKD, we conducted an anonymized online survey before two CKD education outreach sessions to primary care physicians, nurses, and allied health PCPs in public and private practice in October and November 2023. There were 62 responses from physicians and 26 from nurses and allied health staff. Fig. 1 showed that the majority were not comfortable with managing Stage 4 and 5 CKD, including CKD5ND, regardless of the practice type. These results were consistent with recent findings among 266 European PCPs (physicians and nurses), among whom less than half rated themselves fully confident of CKD prognosis and management [3].

**Figure 1:**
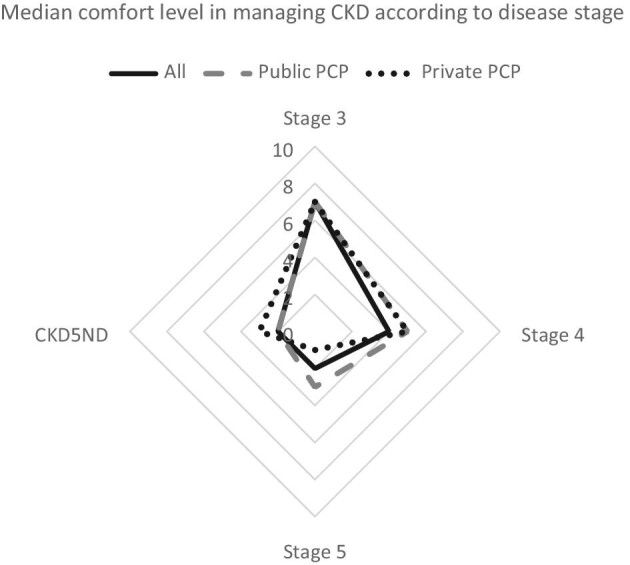
Median comfort level in managing chronic kidney disease (CKD) at different stages, including Stage 5 CKD not on dialysis (CKD5ND). The possible responses were on a scale of 1 to 10, where 1 represented ‘not comfortable’ and 10 represented ‘very comfortable’. There were 88 responses in total, with 42 and 20 responses from primary care physicians in public and private practice, respectively.

To further engage our PCPs, we then evaluated PCP’s preferred methods to improve their comfort in kidney supportive care for CKD5ND. Thirty-nine PCPs in public practice responded to a survey for their preferred Expert Recommendations for Implementing Change (ERIC) strategies in the domains of stakeholder engagement, clinician support, and education and training [4]. Table S1 (see online supplementary material) showed that developing resource sharing agreements, capturing and sharing local knowledge, facilitating relay of clinical data to providers, and conducting educational meetings, outreach visits, and ongoing training, and distributing educational material, were most highly valued by the PCPs. These findings add to prior feedback froms PCPs from other countries that emphasized acccess to educational resources and expertise in conservative or palliative care [2]. More needs to be done to improve kidney supportive care among PCPs and the strategies chosen by PCPs themselves can be used to design PCP facilitation programs that aim to improve the integration of healthcare services for individuals with advanced CKD.

In conclusion, the challenge to manage advanced CKD may not be uncommon among PCPs. Greater understanding of the PCPs’ concerns and successful PCP facilitation programs in different healthcare systems worldwide can provide cross-cultural insights and enable collaborative efforts to enhance kidney supportive care.

## Supplementary Material

sfad305_Supplemental_File
