# Supplementary material for: How primary care physicians view kidney supportive care
Source: Clin Kidney J. 2023 Dec 27;17(1):sfad305. doi: 10.1093/ckj/sfad305 (PMC10802928; doi:10.1093/ckj/sfad305)
Supplement: sfad305_Supplemental_File [file sfad305_supplemental_file.docx]

Supplementary Table S1. Strategies to increase comfort among primary care providers (PCPs) in managing individuals with Stage 5 chronic kidney disease planned for non-dialysis medical therapy. PCPs were allowed to choose more than one option among the Expert Recommendations for Implementing Change (ERIC) implementation strategies in the domains of stakeholder engagement, clinician support and education and training.

|  | Number | Percentage |
| --- | --- | --- |
| **Stakeholder engagement** |  |  |
| - Capture and share local knowledge | 21 | 53.8% |
| - Identify and prepare champions | 10 | 25.6% |
| - Conduct local consensus discussions | 9 | 23.1% |
| - Develop academic partnerships | 8 | 20.5% |
| - Model and simulate change | 8 | 20.5% |
| - Build a coalition | 4 | 10.3% |
| - Visit other sites | 4 | 10.3% |
| - Obtain formal commitments | 3 | 7.7% |
| - Promote network weaving | 3 | 7.7% |
| - Use advisory boards and workgroups | 3 | 7.7% |
| - Involve local opinion leaders | 2 | 5.1% |
| - Involve executive boards | 1 | 2.6% |
| **Clinician support** |  |  |
| - Develop resource sharing agreements | 27 | 69.2% |
| - Facilitate relay of clinical data to providers | 18 | 46.2% |
| - Create new clinical teams | 9 | 23.1% |
| - Revise professional roles | 6 | 15.4% |
| - Remind clinicians | 4 | 10.3% |
| **Education and Training** |  |  |
| - Conduct educational meetings | 16 | 41.0% |
| - Conduct educational outreach visits | 14 | 35.9% |
| - Conduct ongoing training | 13 | 33.3% |
| - Distribute educational materials | 12 | 30.8% |
| - Make training dynamic | 8 | 20.5% |
| - Provide ongoing consultation | 7 | 17.9% |
| - Create a learning collaborative | 5 | 12.8% |
| - Work with educational institutions | 4 | 10.3% |
| - Shadow other experts | 3 | 7.7% |
| - Use train the trainer strategies | 2 | 5.1% |
